# Complete Genome Sequences of Chrysanthemum Stunt Viroid from a Single Chrysanthemum Cultivar

**DOI:** 10.1128/genomeA.00854-15

**Published:** 2015-08-06

**Authors:** Hoseong Choi, Yeonhwa Jo, Ju-Yeon Yoon, Seung-Kook Choi, Won Kyong Cho

**Affiliations:** a Department of Agricultural Biotechnology, College of Agriculture and Life Sciences, Seoul National University, Seoul, Republic of Korea; b Department of Horticultural Environment, Virology Unit, National Institute of Horticultural and Herbal Science, RDA, Wan-Ju, Republic of Korea

## Abstract

The chrysanthemum stunt viroid (CSVd), a member of the genus *Pospiviroid* with a single circular RNA genome, infects many chrysanthemum species. Here, we report 25 complete genome sequences of CSVd in a single chrysanthemum cultivar, revealing 20 variants.

## GENOME ANNOUNCEMENT

Viroids are the smallest pathogens, consisting of a single circular RNA genome, which does not encode any protein (1, 2). The chrysanthemum stunt viroid (CSVd) is a member of the genus *Pospiviroid* in the family *Pospiviroidae* (3, 4). A wide range of chrysanthemum species are major hosts for CSVd, but CSVd also infects other plant species, including *Ageratum*, *Dahlia*, *Petunia hybrida*, and *Senecio* (5). CSVd is mechanically transmitted via grafting and flower cutting. Infection of CSVd in chrysanthemum plants causes chlorotic spots and light green in the leaves as well as stunting (5). Mostly, CSVd infection does not induce visible disease symptoms in infected plants.

Viroids are fast-evolving RNA pathogens and display quasi-species in an infected host plant (6). To obtain information about quasi-species in a single chrysanthemum plant, we screened several commercial chrysanthemum cultivars for CSVd infection by reverse transcription (RT)-PCR using CSVd-specific primers (5′-AAAGAAATGAGGCGAAGAAGTC-3′ and 5′-TTCTTTCAAAGCAGCAGGGT-3′). Of CSVd-infected chrysanthemum cultivars identified, the “Disk club” cultivar showed strong CSVd infection. To obtain information of CSVd quasi-species in the Disk club cultivar, we conducted RT-PCR to amplify the complete genome of CSVd. The obtained PCR products were cloned into the pGEM-T-Easy Vector (Promega, WI, USA), and a total of 25 clones were subjected to sequencing. Out of the 25 complete genome sequences (GenBank accession numbers KT005803 to KT005827) obtained, we found 20 variants. Variant 1, including five CSVd genomes, was the dominant CSVd sequence, followed by variant 2, containing two CSVd genomes. The other 18 variants contain a single CSVd genome. Next, we generated a consensus CSVd genome sequence of 25 CSVd genomes in the Disk club cultivar. Blast search found that the consensus CSVd genome sequence was highly matched to the known CSVd strain SK1 (accession number AB679193.1), with 100% sequence similarity. After alignment of each identified variant sequence to the obtained CSVd consensus genome sequence, we identified 21 single-nucleotide variations. Using complete sequences of 20 variants, a phylogenetic tree was constructed using the MEGA6 program (7). We found that 20 variants were divided into two groups containing 12 and 8 CSVd genomes, respectively.

In summary, we performed CSVd genome sequencing in a single chrysanthemum cultivar, revealing the presence of at least 20 variants. In addition, we demonstrate the quasi-species nature of CSVd with single-nucleotide variations in a single chrysanthemum plant.

### Nucleotide sequence accession numbers.

The genome sequences of the chrysanthemum stunt viroid isolate Disk club have been submitted to GenBank (accession numbers KT005803 to KT005827).
